# Supplementary material for: TWEAK mediates inflammation in experimental atopic dermatitis and psoriasis
Source: Nat Commun. 2017 May 22;8:15395. doi: 10.1038/ncomms15395 (PMC5493595; doi:10.1038/ncomms15395)
Supplement: Supplementary Information — Supplementary Figures and Supplementary Tables [file ncomms15395-s1.pdf]

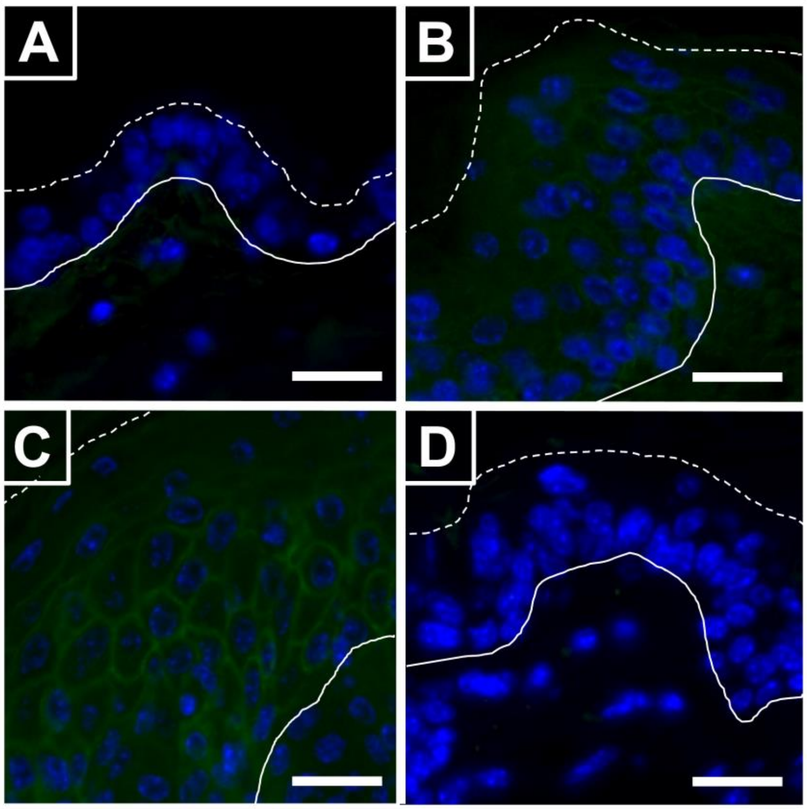

Supplementary Figure 1: Fn14 is upregulated in the epidermis and dermis of mice undergoing AD- and psoriasis-like disease. Immunofluorescence staining for Fn14 (green) and DAPI (blue) in skin of naïve mouse (a), HDM-treated animal (b), and IMQ-treated animal (c). Isotype control staining of IMQ-treated animal in (d). Fn14 signal was seen in the stratum granulosum of the epidermis and in the dermis. Dashed line indicates skin surface, solid line epidermal-dermal junction. Bar is 20 µm.

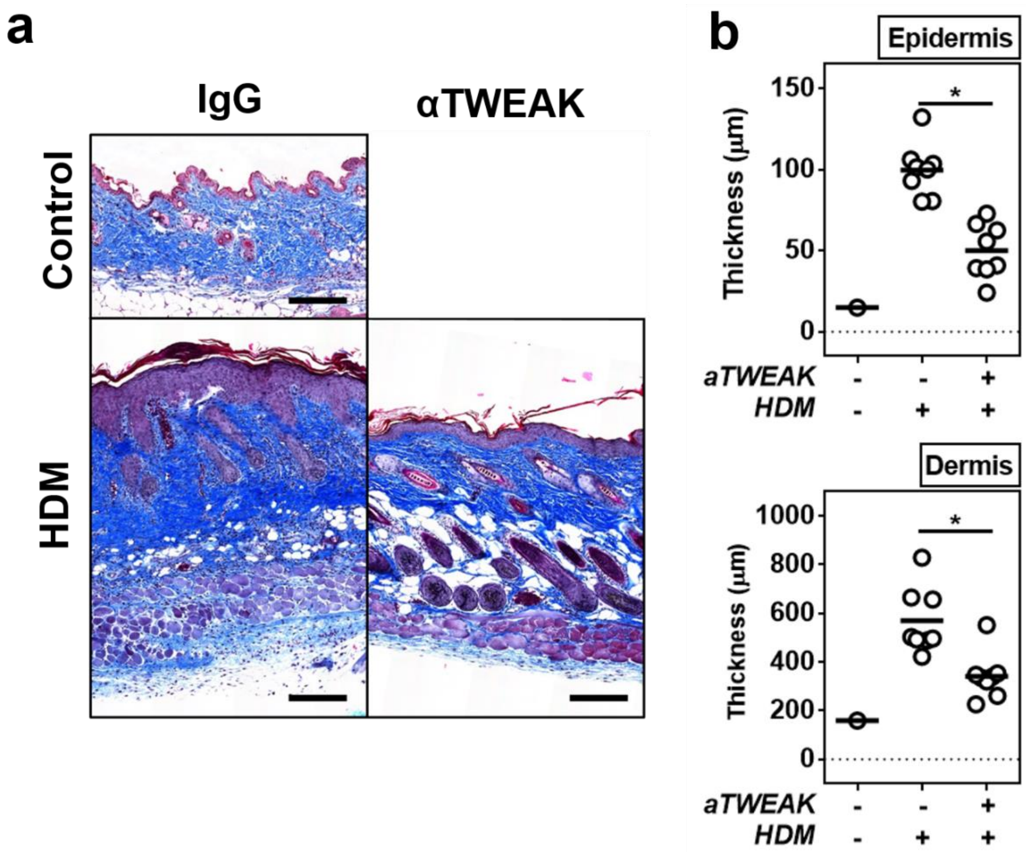

Supplementary Figure 2: Neutralization of TWEAK limits AD disease severity in NC/Nga mice. NC/Nga mice were treated with HDM as in Fig. 1 for 23 days. Starting on day 0, animals were treated with 200 µg anti-TWEAK antibody or IgG control i.p. twice weekly. (a) Masson's trichrome staining of representative skin sections. (b, c) Epidermal and dermal thickness measured from trichrome stained sections. Combined results from two individual experiments with 8 animals per group. Differences among groups were compared using Mann Whitney U test. The Asterix indicates $p < 0.05$.

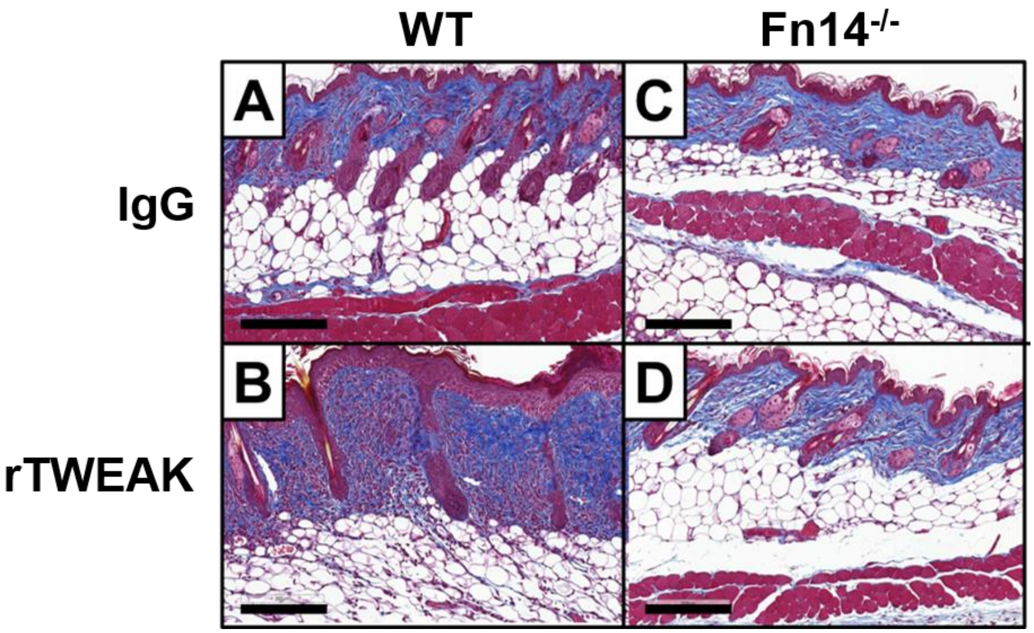

Supplementary Figure 3: Fn14-deficient animals are protected from TWEAK-induced inflammation. Naïve WT or Fn14-deficient mice were injected with 75 µg rTWEAK (b, d) or IgG (a, c), administered s.c. twice weekly over 14 days. Masson's trichrome staining of representative skin sections. Bar represents 200 µm.

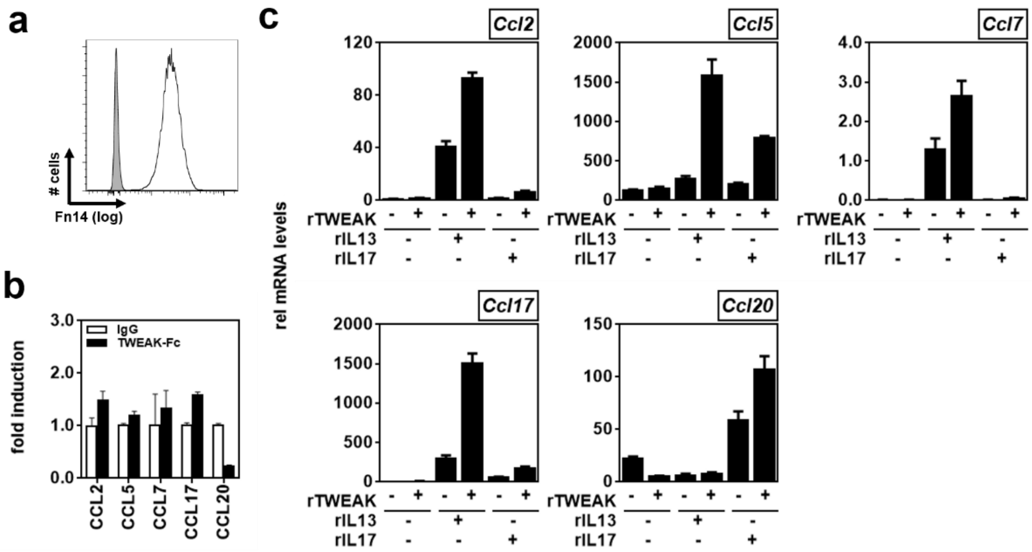

Supplementary Figure 4: Synergistic induction of chemokines by TWEAK, IL-13 and IL-17A in murine keratinocytes. (a) Basal surface expression of Fn14 (line) in PAM212 cells relative to Isotype staining (grey). (b, c) PAM212 cells were stimulated for 48 hours with rTWEAK, rIL-13, rIL-17, or their combination. mRNA expression of indicated chemokines was assessed relative to GAPDH. Means of triplicate cultures with Standard Deviation. One out of three independent experiments.

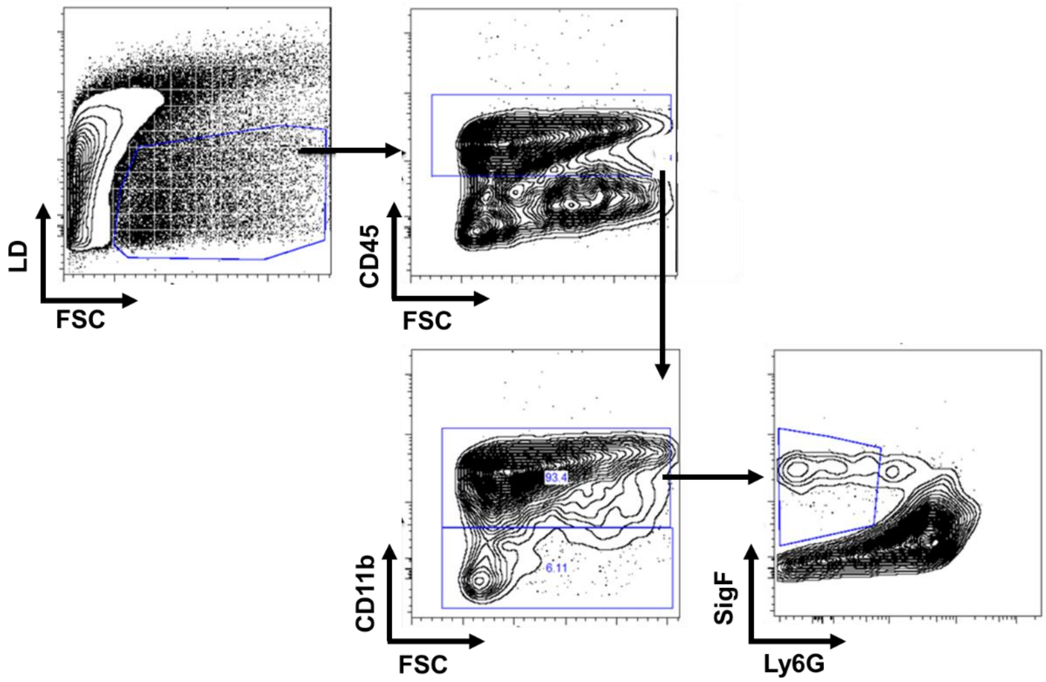

Supplementary Figure 5: Gating Strategy to Quantify Infiltrating Immune Cells. Tissue specimens were digested with Dispase, Collagenase, and DNase, and cells quantified and stained as described in the Materials and Methods. Dead cells were excluded using fixable viability dyes (LD). From live cells, CD45 positive leukocytes were identified, followed by detection of CD11b. Eosinophils were defined as SigF+ and Ly6G negative cells in the CD11b+ fraction.

Supplementary Table 1: Primer Sequences for PCR analysis

**human qPCR primers**	FW (5'- to -3')	RW (5'- to -3')
hCCL2	GTCTCTGCCGCCCTTCTGTG	TCTTTGGGACACTTGCTGCTGG
hCCL5	GGACACCACACCCTGCTGCTTTG	ACACACTTGGCGGTTCTTTCGGG
hCCL7	GCACTTCTGTGTCTGCTGCTC	TGGCTACTGGTGGTCCTTCTG
hCCL17	CTCCTCCTGGGGGCTTCTCT	GTTGGGGTCCGAACAGATGG
hCCL20	GTGCTGTACCAAGAGTTTGCTCC	TGCCGTGTGAAGCCCACAATAAA
hTSLP	TAGCAATCGGCCACATTGCC	CTGAGTTTCCGAATAGCCTG
hIL19	AACCTCCTGGCGTTCTACGTG	TGACTCTGGTGGCATTGGTGG
hGAPDH	TCAACAGCGACACCCACTCCTCC	GGCCATGAGGTCCACCACCCT

**murine qPCR primers**	FW (5'- to -3')	RW (5'- to -3')
mCCL2	ATCCCAATGAGTAGGCTGG	CTCTCTTGAGCTTGGTGACAA
mCCL5	ATGGCTCGGACACCACTCCCTG	GGTTGGCACACACTTGGCGGTT
mCCL7	GCTGCTTTCAGCATCCAAGTGT	ACCGACTACTGGTGATCCTTCTGT
mCCL17	CCGAGAGTGCTGCCTGGATTA	CACAGATGAGCTTGCCCTGGA
mCCL20	TGGGTTTCACAAGACAGATGG	TGAGGAGGTTCACAGCCCTT
mTSLP	TCGAGGACTGTGAGAGCAAGCCAG	GGTAGCCTGGGCAGTGGTCAT
mIL19	CAGCAGCATTGCCAACTCTTTCC	TAAGGGCAGCAGATGAGACCTCC
mGAPDH	AAGAAGGTGGTGAAGCAGG	GAAGGTGGAAGAGTGGGAG
mSDHA	GGAGTGCCGTGGTGTCATTGC	AAGTAGGTTCGCCCGTAGCCC

Supplementary Table 2: Signature Gene Set of the TWEAK/Fn14 Pathway

**Gene Name**	**Description**	**Gene ID**
**CD163**	CD163 molecule	9332
**IKBKB**	inhibitor of kappa light polypeptide gene enhancer in B-cells, kinase beta	3551
**MAP3K14**	mitogen-activated protein kinase kinase kinase 14	9020
**MAPK14**	mitogen-activated protein kinase 14	1432
**MAPK3**	mitogen-activated protein kinase 3	5595
**MAPK8**	mitogen-activated protein kinase 8	5599
**NFKB1**	nuclear factor of kappa light polypeptide gene enhancer in B-cells 1	4790
**NFKB2**	nuclear factor of kappa light polypeptide gene enhancer in B-cells 2 (p49/p100)	4791
**NFKBIA**	nuclear factor of kappa light polypeptide gene enhancer in B-cells inhibitor, alpha	4792
**NFKBIB**	nuclear factor of kappa light polypeptide gene enhancer in B-cells inhibitor, beta	4793
**RAF1**	v-raf-1 murine leukemia viral oncogene homolog 1	5894
**RELA**	v-rel reticuloendotheliosis viral oncogene homolog A (avian)	5970
**RELB**	v-rel reticuloendotheliosis viral oncogene homolog B	5971
**TNFRSF12A**	tumor necrosis factor receptor superfamily, member 12A	51330
**TRAF1**	TNF receptor-associated factor 1	7185
**TRAF2**	TNF receptor-associated factor 2	7186
**TRAF3**	TNF receptor-associated factor 3	7187
